# Saxitoxin-Producing *Raphidiopsis raciborskii* (Cyanobacteria) Constrains *Daphnia* Fitness and Feeding Rate despite High Nutritious Food Availability

**DOI:** 10.3390/toxics11080693

**Published:** 2023-08-11

**Authors:** Gabriele Costa dos Reis, Gustavo Henrique A. de Carvalho, Mauro Cesar Palmeira Vilar, Sandra Maria Feliciano de Oliveira e Azevedo, Aloysio da Silva Ferrão-Filho

**Affiliations:** 1 Laboratory of Evaluation and Promotion of Environmental Health, Instituto Oswaldo Cruz, FIOCRUZ, Rio de Janeiro 21040-360, Brazil; gabrieledreis@gmail.com (G.C.d.R.); gustavostew@gmail.com (G.H.A.d.C.); 2 Laboratory of Ecophysiology and Toxicology of Cyanobacteria, Carlos Chagas Filho Institute of Biophysics, Federal University of Rio de Janeiro, Rio de Janeiro 21949-902, Brazil; maurovilar@biof.ufrj.br (M.C.P.V.); sazevedo@biof.ufrj.br (S.M.F.d.O.e.A.)

**Keywords:** zooplankton, cyanotoxins, nutritional constraint, cyanobacteria

## Abstract

Changes in food quality can dramatically impair zooplankton fitness, especially in eutrophic water bodies where cyanobacteria are usually predominant. Cyanobacteria are considered a food with low nutritional value, and some species can produce bioactive secondary metabolites reported as toxic to zooplankton. Considering that cyanobacteria can limit the survival, growth and reproduction of zooplankton, we hypothesized that the dietary exposure of neotropical *Daphnia species* (*D. laevis* and *D. gessneri*) to saxitoxin-producing cyanobacteria impairs *Daphnia* feeding rates and fitness regardless of a high availability of nutritious algae. Life table and grazing assays were conducted with different diets: (1) without nutritional restriction, where neonates were fed with diets at a constant green algae biomass (as a nutritious food source), and an increasing cyanobacterial concentration (toxic and poor food source), and (2) with diets consisting of different proportions of green algae (nutritious) and cyanobacteria (poor food) at a total biomass 1.0 mg C L^−1^. In general, the presence of high proportions of cyanobacteria promoted a decrease in *Daphnia* somatic growth, reproduction and the intrinsic rate of population increase (*r*) in both diets with more pronounced effects in the nutritionally restricted diet (90% *R. raciborskii*). A two-way ANOVA revealed the significant effects of species/clone and treatments in both assays, with significant interaction between those factors only in the second assay. Regarding the grazing assay, only *D. laevis* was negatively affected by increased cyanobacterial proportions in the diet. In the life table assay with constant nutritious food, a reduction in the reproduction and the intrinsic rate of the population increase (*r*) of all species were observed. In conclusion, we found adverse effects of the toxic cyanobacterial strain *R. raciborskii* on *Daphnia* fitness, regardless of the constant amount of nutritious food available, proving the toxic effect of *R. raciborskii* and that the nutritional quality of the food has a greater influence on the fitness of these animals.

## 1. Introduction

Factors such as intensified industrialization, urban growth and agricultural activities lead to increased inputs of nitrogen and phosphate nutrients in aquatic ecosystems, causing eutrophication [1]. This process has favored the occurrence of cyanobacterial harmful algal blooms (CyanoHABs) which have been boosted by climate changes [2]. Bloom-forming cyanobacteria can cause negative impacts in aquatic ecosystems by cyanotoxins production, oxygen depletion, shading of benthic habitats and trophic decoupling [3,4,5].

Cyanobacteria are photosynthetic prokaryotes that usually compose marine and freshwater phytoplankton, and contribute to the primary production in aquatic food webs as a significant food source for zooplankton consumers, especially in eutrophic water bodies [6,7,8]. However, dietary exposure to cyanobacteria can affect zooplankton due to its low nutritional quality, such as the lack of polyunsaturated fatty acids (PUFAs) and sterols [9], difficult digestibility [10] or by the ability of some cyanobacterial genera on producing bioactive secondary metabolites (cyanotoxins) which can affect zooplankton motility, feeding behavior, survival and life cycles [11,12].

Among cyanobacteria, *Raphidiopsis raciborskii* (formerly *Cylindrospermopsis raciborskii*) [13] stands out as a N-fixing and bloom-forming species which has been recently reported for higher latitudes as an invasive aquatic microbe. The ability of *R. raciborskii* on spreading over several regions is attributed to its high phenotypic plasticity regarding tolerance to light, salinity, ability to store phosphorus, toxin production and resistance to herbivory [14,15,16]. Toxin-producing strains of this species can produce cytotoxic (cylindrospermopsins) [17,18,19] and neurotoxic (saxitoxins, goniautoxins and C toxins, hereafter STXs) alkaloids; the latter toxins being produced only by south American strains to date [20,21,22,23,24]. *R. raciborskii* blooms are increasingly frequent in tropical lakes and reservoirs which makes this species a representative component of phytoplankton communities in these water bodies [25,26,27].

Overall, the establishment of cyanoHABs usually represents a change from diverse and edible phytoplankton to a less diverse and inedible one (large colonies and filaments, and the production of harmful metabolites) [5,28]. In turn, it can affect zooplankton composition, which shifts from a community dominated by large generalist filter feeders such as cladocerans (e.g., *Daphnia*), toward small-bodied selective organisms such as copepods, rotifers and planktonic protozoans [5,29]. However, although there is a consensus that cyanobacteria play a role in the trophic decoupling of freshwater food webs [30], mainly due to defensive chemical traits against phytoplankton consumers [16,31], little is known whether the increased cyanobacterial biomass affects the zooplankton due to a relatively low abundance of nutritious algae or if cyanobacterial toxicity overrides nutritional constraints regardless of high-quality food availability at a non-limiting biomass concentration. Therefore, we evaluated whether the negative impacts of dietary exposure of *Daphnia* to toxic cyanobacteria are mostly driven by nutritional constraints due to nutritious algae availability. We tested the hypothesis that the toxicity of STXs-producing *R. raciborskii* impairs *Daphnia* feeding rates and fitness regardless of the high availability of nutritious algae.

## 2. Methods

### 2.1. Phytoplankton and Zooplankton Culture Conditions

The saxitoxin-producing strain *Raphidiopsis raciborskii* CYLCAM-01 [32] was kindly provided by Dr. Marcelo M. Marinho (Laboratory of Ecology and Physiology of Phytoplankton—Rio de Janeiro State University). The cyanobacterium was isolated from the Camorim reservoir; a eutrophic water body located at the Pedra Branca State Park (PEPB)—Rio de Janeiro, Brazil. The strain was cultivated in a WC medium [33] with initial pH adjusted to 8.0 and maintained at a temperature of 23 ± 1 °C, a light intensity of 50 μmol m^−2^s^−1^ and a 12:12 h light/dark photoperiod.

The tested zooplankton consisted of a neotropical *Daphnia* species isolated from different Brazilian water bodies: *D. laevis* (Lagoa do Ibirité-Minas Gerais), *D. laevis* (Lagoa Jacaré-Minas Gerais) and *D. gessneri* (Reservatório do Apertado-Bahia). Animals were kept in 1000 mL beakers filled with artificial RT medium [34] enriched with 30% filtered (filter 1–2 µm pore size, Sartorius) natural water from decantation tanks of the CEDAE treatment plant (State Company for Water and Sewage, Camorim Reservoir) and supplied with commercial 0.05% humic acid extract (~1.125 mg C L^−1^, Microbe Lift^®^ Amazon Black & Soft Water Conditioner, USA) at an initial pH 7.6. The animals were incubated at 23.5 ± 1 °C under low light intensity (20 μmol m^−2^s^−1^) and a 12:12 h light/dark photoperiod. To feed the animals, the green microalgae *Monoraphidium capricornutum* and *Ankistrodesmus stiptatus* were cultivated in oligo medium [35] with the initial pH adjusted to 7.0. For cultures maintenance, the animals are usually fed every two days with a total carbon biomass of 0.4 mg L^−1^ for both algal species (1:1).

### 2.2. Phytoplankton Biomass Estimative and Saxitoxin Analysis

Cyanobacterial filaments and microalgal cells were counted in a Fuchs-Rosenthal chamber under an optical microscope to determine the cell/filament density (unit mL^−1^). Subsequently, the population density was converted to biovolume (mm^3^ L^−1^) from the average cell/filament volume (µm^3^) [36,37] obtained by cells and filaments dimension (length and width) measurements under an optical microscope with the aid of CellB software (Soft Imaging System GmbH, Münster, Germany). Finally, the carbon biomass (mgC L^−1^) was estimated from the cell volume measurements as described by Rocha and Duncan [38].

STX was analyzed by Enzyme-Linked Immunoabsorbent Assay (ELISA) using a Beacon saxitoxin kit (Beacon Analytical Systems, Inc., Saco, ME, USA) according to the manufacturer’s recommendation.

### 2.3. Life Table Experiments

Life table experiments were run to evaluate whether the amount of saxitoxin-producing cyanobacteria in the diet could affect zooplankton fitness, biomass acquisition and survivorship, despite the availability of nutritious green microalgae (hereafter nutritious food) as follows:

### 2.4. Dietary Exposure to STX-Producing Cyanobacteria under Constant Nutritious Food

In the first experiment, neonates (<24 h) of the species *D. laevis* (RD clone), *D. laevis* (IB clone) e *D. gessneri* were exposed to increasing concentrations of the toxic *R. raciborskii* CYLCAM-01 (0, 0.5, 1.0 and 1.5 mg C L^−1^) and a constant biomass concentration of nutritious food (0.4 mg C L^−1^ of *M. capricornutum*) over 15 days. The experiment was run with one individual per test tube (n = 15) filled with 30 mL of the phytoplankton cell suspensions at the different biomass concentrations (experimental diets) and incubated over 15 days. We aimed to keep a constant nutritious food concentration to rule out effects due to nutritional deficiency caused by the lack of important constituents in cyanobacteria for zooplankton development, such as polyunsaturated fatty acids and sterols, which green algae can provide [9], and to check if the toxicity would still hold.

### 2.5. Dietary Exposure to STX-Producing Cyanobacteria under Variable Nutritious Food

In the second experiment, neonates (<24 h) from each *Daphnia* species/clone were exposed to increased cyanobacterial proportions (25–90%) relative to nutritious food (75–10%). For the nutritious food (green algae), we kept a higher proportion of *A. stiptatus* (90%) relative to *M. capricornutum* since this microalgae seems to be more suitable for zooplankton nutrition regarding its lipid composition (as well as phosphate content) which consists mainly of unsaturated fatty acids (C18 chains such as alpha-linolenic acid, oleic acid and linoleic acid) [39]. Thus, the *Daphnia* dietary exposure to toxic *R. raciborskii* CYLCAM-01 was set into three experimental diets: (i) 25% CYLCAM-01 + 75% nutritious food; (ii) 50% CYLCAM-01 + 50% nutritious food; and (iii) 90% CYLCAM-01 + 10% nutritious food. The control group consisted of 100% nutritious food. All proportions were relative to a total carbon biomass concentration of 1.0 mg C L^−1^. The experiment was run in test tubes (n = 15) filled with 30 mL of phytoplankton cell suspensions and incubated over 15 days. For all experimental diets, the medium was renewed daily over the experiment duration. This experiment differed from the previous one as the concentration of nutritious food varied, but the total amount of phytoplankton was constant (Table 1). This was supposed to test for the effect of a more restricted diet at higher proportions of cyanobacteria.

For the life table parameters, neonates individually exposed to the different diets were assessed from newborn to maturity during 15 days or up to at least the third brood, to assess the (*i*) age at first reproduction (primiparous) and the number of neonates produced per female in each brood. The survival (*l_x_*) and mean fecundity (*m_x_*) were used to estimate the mean intrinsic rate of population increase (*r*) by bootstrap analysis (Software Rm 2.0 [40]) using the following equation:

$$1=\sum l_{x}m_{x}e^{-rx}$$

where *l_x_* is age-specific survival, *m_x_* is age-specific fecundity and *x* is the age in days.

At the beginning and the end of the experiments, the organisms of each treatment were measured for body length from the top of the head to the base of the caudal spine under an Olympus magnifying glass (SZ61) with 20× magnification to estimate the somatic growth rate by the following formula:

$$\frac{\left(lnL_{f}\right)-\left(lnL_{i}\right)}{\Delta t}$$

where *L_i_* is the initial length, *L_f_* is the final length and *t* is the incubation time in days.

### 2.6. Grazing Assays

Grazing assays were performed to evaluate whether an increased proportion of saxitoxin-producing cyanobacteria in the diet, at a constant non-limiting biomass concentration, did or did not affect the zooplankton feeding rate.

Adult individuals (~6 days) of all *Daphnia* clones were exposed to phytoplankton cell suspensions via their diet at increased proportions of *R. raciborskii* CYLCAM-01 (25, 50 and 90%) relative to the nutritious food (=green microalgae *M. capricornutum*), at a total carbon biomass concentration of 1.0 mg C L^−1^. The animals were starved (~12 h) before the beginning of the experiment and then conditioned in 10 mL test tubes (n = 4; 2 individuals/test tube) filled with the different experimental diets. A cellular suspension composed of 100% nutritious food was assumed as the negative control for cyanobacterial impact by dietary exposure. Alongside this, all experimental diets were also established without animals as the negative control for cell loss by *Daphnia* grazing.

The *Daphnia* clones were incubated in the dark for 3 h and, subsequently, the clearance rate (CR, in mL ind^−1^ h^−1^) was estimated from chlorophyll fluorescence (ChlF0) emitted from phytoplankton cells in the experimental diets and detected by a PHYTO-PAM fluorimeter (Walz, Germany). The clearance rate was estimated according to Lürling and Verschoor [41] using the following equation:

$$CR=\frac{\left\{ln\left(Chla_{control})-ln(Chla_{treatment}\right)\right\}}{∆t}\times \frac{V}{N}$$

with *Chla_control_* and *Chla_treatment_* as the difference in the algal concentration (as chlorophyll-*a* specific for Cyanobacteria and green algae; µg L^−1^) at the initial and final time in the control (without animals) and treatments (with animals), respectively. Δ*t* is the incubation time (*h*), *V* is the volume of culture medium (mL) and *N* is the number of animals.

The calculation of clearance rate was based on the fluorescence signals given: green (for green algae), blue (for cyanobacteria) and total chlorophyll (for the sum of both signals).

### 2.7. Data Analysis

CL_50_ (Lethal Concentration to 50% of individuals) and CE_50_ (Effective Concentration where there is a 50% decrease in reproduction) were estimated in the online platform MOSAIC [42].

Population parameters (reproduction parameters and the intrinsic rate of population increase (*r*)), as well as the feeding rate were examined for normality and variance homoscedasticity. Once the parametric premises were assumed, the data were compared using a one-way ANOVA, and the differences between treatments were analyzed by the Tukey HSD test (*p* < 0.05) using the GraphPad Prism 5.0 software. To verify the differences regarding the population parameters between *Daphnia* species/clones and treatments, we used the two-way ANOVA, with species/clone and treatment as the categorical factors. The intrinsic rate of population increase (*r*) was analyzed by Student’s *t*-test (*p* < 0.05), using the Primer 1.0 program.

## 3. Results

### 3.1. Dietary Exposure to STX-Producing Cyanobacteria under Constant Nutritious Food

**Figure 1:**
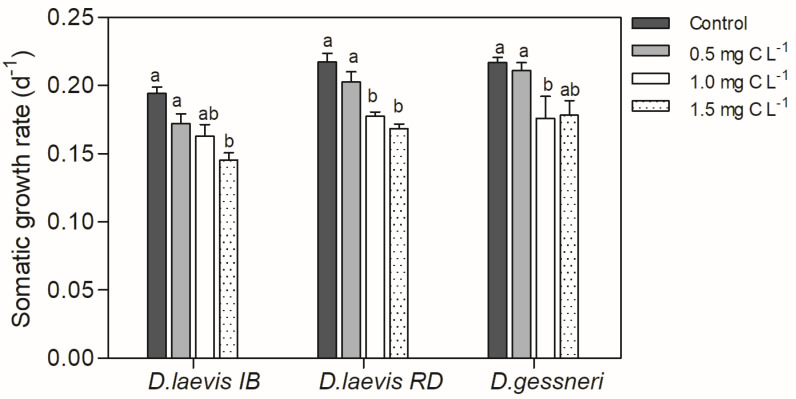
Somatic growth rate of *D. laevis* (IB), D. laevis (RD) and *D. gessneri* in assay 1, exposed to cyanobacteria and without nutritional restriction (constant concentration of green algae at 0.4 mg C L^−1^). Control refers to a concentration of only green algae (0.4 mg C L^−1^). Different letters indicate significant differences (ANOVA, Tukey test *p* < 0.05). Standard error bars are given.

In this assay, the animals were exposed to a cell-bound STX concentration ranging from 1.5 to 4.5 ng L^−1^ as shown in Table 1. Only *D. gessneri* showed reduced survival by the increasing concentrations of cyanobacteria, with a reduction in the number of individuals throughout the experiment. The estimates of LC_50_ and CE_50_ are shown in Table 2. No effects were observed on the survival of *D. laevis* clones. There was a significant reduction in the somatic growth rate of all species tested (Figure 1).

The two-way ANOVA revealed a significant effect of species/clone and treatment in all population parameters, with no interaction between those factors (Table 3).

**Figure 2:**
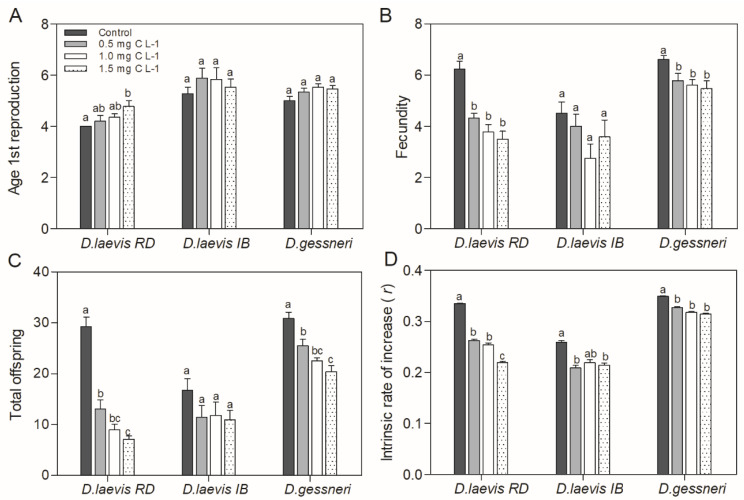
Population parameters of *D. laevis* (RD), *D. laevis* (IB) and *D. gessneri* in assay 1, without nutritional restrictions. (**A**) Age of first reproduction; (**B**) fecundity; (**C**) total offspring; (**D**) intrinsic rate of population increase (*r*). Treatments have a constant concentration of green algae (0.4 mg C L^−1^) and a variable concentration of cyanobacteria. Control refers to a concentration of only green algae (0.4 mg C L^−1^). Different letters indicate significant differences (ANOVA, Tukey’s test, *p* < 0.05). Standard error bars are given.

Despite the constant amount of nutritious food, the presence of STX-producing cyanobacteria in the diet promoted adverse effects on *Daphnia* populational parameters such as the age at first reproduction, fecundity, total offspring and intrinsic population growth rate (*r*) of all species (Figure 2). In the parameter age of first reproduction, a significant delay in reproduction (ANOVA, Tukey test, *p* = 0.009) was observed only for *D. laevis* (RD) at the highest concentration of cyanobacteria (Figure 2A). In the average fecundity, only *D. laevis* (RD) and *D. gessneri* suffered a significant negative effect with increasing concentrations of cyanobacteria in the diet (Figure 2B). The total number of neonates produced by *D. laevis* (RD) and *D. gessneri* were reduced significantly at all concentrations, with a greater impact on the *D. laevis* (RD) clone. Although the clone *D. laevis* (IB) was not significantly affected in the parameters of mean fecundity and total neonates produced in any of the treatments (Figure 2B,C), there was a reduction in the intrinsic rate of population increase (*r*) of this clone as well as in the other clones (Figure 2D). Again, the clone *D. laevis* (RD) was most affected, with reductions of *r*-value in the order of 21 to 33%.

### 3.2. Dietary Exposure to STX-Producing Cyanobacteria under Variable Nutritional Food

The analysis detected STX concentrations ranging from 5.0 to 15.2 ng L^−1^ (Table 1). As in the previous life table assay with fixed nutritious food, *D. laevis* clones showed no reduction in survival. On the other hand, *D. gessneri* experienced a reduction in survival over the 15 days of exposure, with a concentration-dependent decline in the number of individuals and mortality of all individuals in the 90% *R. raciborskii* treatment from day 11 and the 50% treatment on day 14. The LC_50_ and EC_50_ values can be seen in Table 4.

For *D. gessneri*, a dietary exposure to 25% of cyanobacteria caused a 50% reduction in survivorship, while about 56% cyanobacteria in the diet caused a 50% reduction in the production of offspring (Table 4). For both *D. laevis* clones, this effect on reproduction was caused at a higher proportion of cyanobacteria in the diet (81 and 89%, respectively; Table 4).

**Figure 3:**
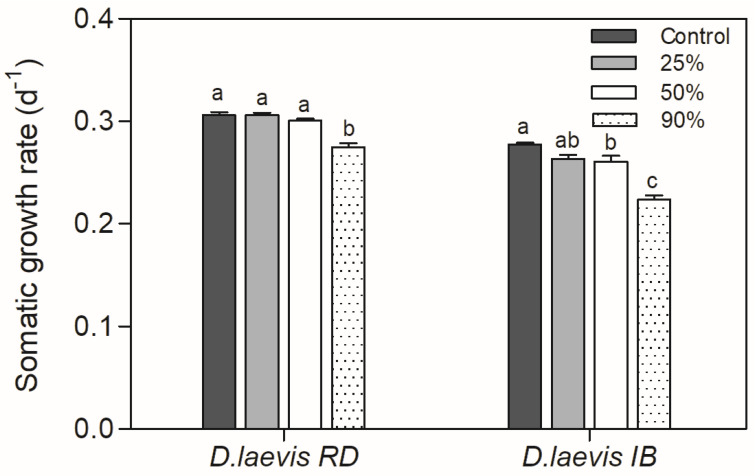
Somatic growth rate of *D. laevis* (RD) and *D. laevis* (IB) in assay 2, exposed to treatments with different proportions of cyanobacteria (25–90%) and green algae (75–10%) at a total concentration of 1.0 mg C L^−1^. Control refers to 100% green algae. Different letters indicate significant differences (ANOVA, Tukey test *p* < 0.05). Standard error bars are given.

A significant reduction in the somatic growth rate was observed in the treatment with 90% cyanobacteria in the diet (0.9 mg C L^−1^) for *D. laevis* (RD) and 50% and 90% (0.5 and 0.9 mg C L^−1^) for *D. laevis* (IB) (*p* > 0.0001) (Figure 3). It was not possible to calculate the growth rate for *D. gessneri* once all organisms died in the treatments with cyanobacteria prior to end of the life table experiment.

Overall, the two-way ANOVA revealed a significant effect of species/clone and treatment only for fecundity as well as a significant interaction between those factors (Table 5). The other parameters showed a significant effect only for treatment, besides an interaction between those factors (Table 5).

**Figure 4:**
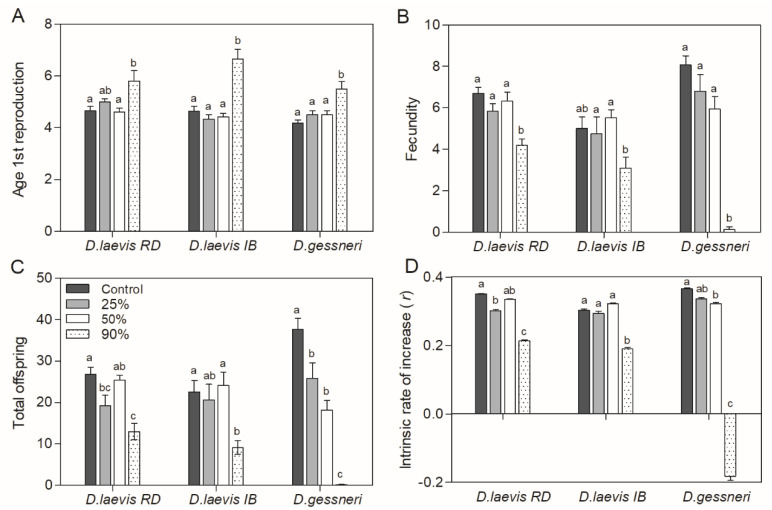
Population parameters of *D. laevis* (RD), *D. laevis* (IB) and *D. gessneri* in assay 2, with different proportions of green algae (100–10%) and cyanobacteria (0–90%) at a total concentration of 1.0 mg C L^−1^. Control refers to 100% green algae. (**A**) Age of first reproduction; (**B**) fecundity; (**C**) total offspring; (**D**) intrinsic rate of population increase (*r*). Different letters indicate significant differences (ANOVA, Tukey’s test, *p* < 0.05). Standard error bars are given.

Population parameters as well as the somatic growth rate were negatively affected only at 90% *R. raciborskii* for all clones, with *D. gessneri* presenting a greater impact in the total offspring production and in the intrinsic rate of population increase (*r*-values) (Figure 4). Likewise, total offspring was significantly reduced only at the 90% treatment for *D. laevis* (RD; *p* < 0.001) and *D. laevis* (IB; *p* = 0.006), and for *D. gessneri p* < 0.001 there was a more drastic reduction in total offspring, also being significant in the 25% and 50% *R. raciborskii* treatments (Figure 4C). The intrinsic rate of population increase (*r*) was indeed negative for *D. gessneri* on the 90% cyanobacterial diet (*p* < 0.05), due to the reduced number of neonates produced per female (only one female reproduced in this treatment) (Figure 4D).

### 3.3. Cyanobacterial Effects on Feeding Rate

**Figure 5:**
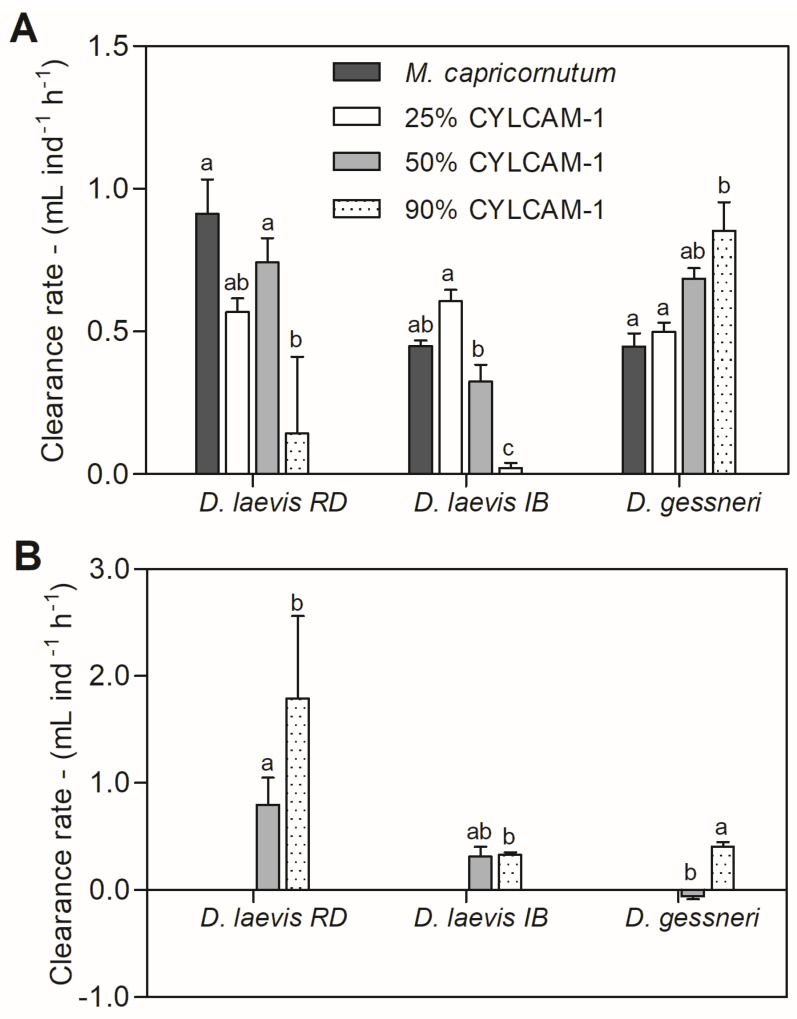
(**A**) Clearance rate of *M. capricornutum* and (**B**) clearance rate of *R. raciborskii* of *D. laevis* (RD), *D. laevis* (IB) and *D. gessneri* exposed to treatments with varying proportions of green algae (100–10%) and cyanobacteria (0–90%) at a total concentration of 1.0 mg C L^−1^. Different letters indicate significant differences (ANOVA, Tukey test *p* < 0.05). Standard error bars are given.

The animals showed a significant reduction in the clearance rate of the green algae for both *D. laevis* clones in the 90% treatment (Figure 5A). *D. gessneri* had a totally different behavior, with an increase in the clearance rate of *M. capricornutum* with increasing concentrations of cyanobacteria (Figure 5A). When only the cyanobacterial signal was analyzed, with the exception of the *D. laevis* (RD) clone, clearance rates were in general lower than the green algae. However, significant reductions were observed only in the 90% treatment for *D. laevis* (IB) and in the 50% cyanobacterial diet for *D. gessneri* (Figure 5B).

No cyanobacterial signal was detected in the 25% treatment for *D. laevis* (RD) and *D. gessneri* so it was not possible to quantify the cyanobacterial clearance rate at this concentration for those species.

## 4. Discussion

The results corroborated our main hypothesis that the toxicity of STX-producing *R. raciborskii* impairs *Daphnia* feeding rates and fitness regardless of the high availability of nutritious algae. This effect was, however, species/clone dependent. In both life table assays, there was a reduction in the somatic growth and population increase rates of all species exposed to cyanobacterial concentrations relative to the control. It is probable that the energetic cost invested in survival may have decreased the fitness of these animals, causing reduced fecundity and growth. The quality of food is determined by a variety of substances in natural environments. Cyanobacteria are generally considered a poor food due to a lack of specific micronutrients, such as polyunsaturated fatty acids and sterols [9,43]. If diets are deficient in the essential substances necessary for growth, such as sterols and fatty acids, growth rates may be affected [44]. However, species/clones may respond differently to the variable diets, according to their sensitivity and nutritional requirements [45,46].

The two experimental designs had some important differences that should be pointed out. In the first one, our purpose was to exclude the nutritional effect by providing a non-limiting amount of nutritious food (green algae) at a constant ratio while varying the concentration of cyanobacteria (0 to 1.5 mg C L^−1^); thus, the total food concentration varied (from 0.4 to 1.9 mg C L^−1^) and so the proportion of nutritious food in the diet varied (100 to 21%). The second one was performed with variable proportions between cyanobacteria and green algae so the total food concentration remained constant at 1.0 mg C L^−1^. Therefore, the equivalent concentration of cyanobacteria in both assays do not represent the same proportion of the total food in each assay. For example, at 1.0 mg C L^−1^ of cyanobacteria in assay 1 there was ~30% of nutritious food, while at 0.9 mg C L^−1^ in assay 2 there was only 10% of nutritious food (see Table 1). Thus, at the equivalent cyanobacterial concentrations, nutritional restriction was much higher in assay 2.

The negative effects on the reproductive parameters and somatic growth of *D. laevis* (RD) were more pronounced in the experiment without nutritional restrictions. Therefore, the low nutritional value of cyanobacteria alone does not explain the reduction in fecundity and somatic growth, since these organisms received a sufficient amount of food with high nutritional quality, probably evidencing a toxic effect due to the presence of STX. In addition, at equivalent concentrations of cyanobacteria (~1.0 mg C L^−1^) there was a higher concentration of nutritious food in assay 1 than in assay 2 which corroborates this hypothesis. In a previous study, a *D. laevis* clone isolated from Lagoa da Pampulha (MG, Brazil) showed reduced fecundity when chronically exposed to a CYRF-1 strain (STXs producer) and NPCS-1 strain (non-STXs producer), both strains of the species *R. raciborskii*. However, the effects of the CYRF-1 strain were more pronounced than those of the NPCS-1 strain, showing that besides toxicity, nutritional constraints must play a role on the reduced fecundity [47].

On the other hand, in the life table assay with variable nutritional food availability, *D. laevis* clones did not show much difference in reproductive parameters, both being equally affected only in a high share of cyanobacteria (90%). At a lower share of *R. raciborskii* (25–50%), *D. laevis* seems to cope with the presence of this poor food resource, showing good growth rates. This species can coexist for long periods with cyanobacterial blooms, but its total lipid reserves decrease [48], which could explain the reduced fitness of these animals when they were exposed to treatments with higher proportions of cyanobacteria. In studies with cylindrospermopsin-producing (*R. raciborskii* and *Aphanizomenon ovalisporum*) and non-producing (*R. raciborskii*) strains, effects on the survival and somatic growth of *D. magna*, as well as damage to the digestive epithelium, were also observed [49].

Among the species, *D. gessneri* showed the most contrasting responses between the two life table assays performed. While in the assay without nutritional restrictions *D. gessneri* was the most tolerant among the species tested, in the trial with the nutritional restriction it suffered the most deleterious effects on reproduction, having extremely low fecundity and total offspring production, and a negative rate of population increase in the highest proportion (90%) of cyanobacteria in the diet. Regarding survival, in both life table assays, the results did not show any resistance of *D. gessneri* as in previous studies where this species showed high survival. Using the *R. raciborskii* T3 strain (STXs producer), authors found that it did not interfere with the survival or reproduction of *D. gessneri*, while the cladocerans *D. pulex* and *M. micrura* had reductions in their survivorship and population increase rate [50]. Therefore, it is possible that *D. gessneri* is more tolerant to toxins and sensitive to a lack of nutritious food.

Using mixtures of cyanobacteria and green algae (*Scenedesmus obliquus*), effects of the CYRF-1 strain on *D. magna*, such as reductions in survival, body growth, population growth rate and clearance rate, have been reported [51]. As for *D. laevis* in our experiment, a proportion of 1:1 (50:50%) between green algae and cyanobacteria was enough to reverse the effects of the CYRF strain on *D. magna*. In an assay with *D. pulicaria* being exposed to treatments containing *S. obliquus* or filaments of a non-toxic strain of *R. raciborskii*, the presence of a non-toxic strain affected the reproductive success of *Daphnia*, not only through reduced fecundity but also through an increase in the number of aborted eggs [52]. This study, as well as that of Restani and Fonseca [47] with the non-toxic strain NPCS-1, reinforces the fact that the observed effects on reproduction are not only explained by the toxins and nutritional issues are involved.

In a study with another *R. raciborskii* strain (CYLCAM-2) isolated from the same reservoir and with the same experimental design of our study, but with more limited food conditions (total food = 200 mg C L^−1^), the four cladocerans tested showed decreased fitness even in the lower proportion of cyanobacteria (25%), with differences in sensitivity between the species/clones [46]. Apparently, *D. laevis* (IB clone) was one of the most resistant species being more affected only at the highest proportion of cyanobacteria in the diet (100%). Thus, this clone seems to be more affected by nutritional deficiency than toxicity. In the present study, *D. gessneri* showed to be very sensitive to a lack of nutritious food, having its fitness severely depressed in the highest proportion of cyanobacteria (90% of the diet).

The grazing assay showed a decreased total clearance rate only for the *D. laevis* (IB) clone when exposed to a diet with cyanobacteria. When the clearance rate of green algae was analyzed, there was a significant reduction for both *D. laevis* clones. Those clones probably reduced their clearance rate due to the presence of toxins, poor nutritional quality and/or morphology of *R. raciborskii*. In a previous study, *D. magna*, even presenting feeding inhibition, continued to have a positive population increase rate (*r*), indicating that these organisms can thrive in eutrophic environments dominated by cyanobacteria [51]. Similarly, it is possible that despite the reduction in clearance rate, *D. laevis* maintained its fitness, since in the life table assay with variable food availability both clones were less affected in reproductive parameters. Curiously, the clearance rate of cyanobacteria for the clone RD was higher in the 90% treatment, showing that this clone is capable of ingesting high amounts of cyanobacterial filaments. This may have compensated the decreased ingestion of green algae.

It has been suggested that filaments (with an average length of 426.7 ± 208.4 µm in this study) could impair the ingestion of food by *Daphnia* [53,54,55]. Although the *D. laevis* clones are medium-sized (1.8 mm), the ingestion of large filaments has been observed in other studies with cladocerans of a similar size. *D. pulex* (0.6 mm), when exposed to STX-producing strains, was significantly more affected in its clearance rate by ingesting long filaments of MVCC19 (863.5 ± 483.3 µm) and CYRF-01 (338.7 ± 190.3 µm) than the copepod *Notodiaptomus iheringi* (1.1 mm) [56]. Copepods seem to cope better with the presence of filamentous cyanobacteria [57]. They are able to cut long filaments [58] and can avoid toxic strains [59]. Cladocerans, however, are generalist filter-feeders and filamentous cyanobacteria can clog their filtering apparatus [60]. Nevertheless, *D. laevis* (RD) seems to ingest the filaments of *R. raciborskii*, becoming more exposed to the toxins of the strain. This is the reason it may have been more affected in the life table experiment with no food restriction. In addition, the toxin production of *R. raciborskii* can also reduce the fitness of copepods, especially when exposed to nutritionally restricted diets, indicating that toxins interfere more with herbivory than filament size [31,61].

In the grazing assay, *D. gessneri* showed a tendency to increase the total clearance rate in the treatments with cyanobacteria, which may be attributed to an increase in the ingestion of green algae. On the other hand, this species showed a reduced clearance rate of cyanobacteria in the 50% treatment, but a comparable rate to green algae in the 90% treatment, which may explain its reduced fitness, as it may be able to ingest the poor food in an environment with low nutritious food availability. DeMott [62] has shown that *Daphnia* spp. exposed to toxic *Microcystis* exhibited inhibition of feeding after 1 h of exposure to toxic food, but had substantially recovered after 24 h in the same mixtures (“hunger effect”). Thus, it is possible that *D. gessneri* elevated its feeding rate in the 90% cyanobacterial diet to decrease the risk of starvation, as the availability of nutritious food was too low.

Some studies have already demonstrated selectivity in cladocerans. Although in general they are considered non-selective filter-feeders, experiments with *D. ambigua* revealed that they can avoid toxic strains of *M. aeruginosa* [63]. Using video recording, *D. pulicaria* was able to discriminate between toxic *Microcystis* strains and the green algae *Scenedesmus*, slowing its mandible movement in the presence of this cyanobacterium [64]. Higher clearance rates of the green algae *S. capricornutum* were observed in treatments with a mixture of *Microcystis aeruginosa* (MIRF-1) and *R. raciborskii* (CYRF-1) than in all other treatments for *D. laevis* and *D. similis*, suggesting no feeding inhibition of these cladocerans and perhaps selective clearance [65]. Therefore, the possibility that *D. gessneri* is able to discriminate toxic cyanobacteria at the same time that it increases its clearance rate of green algae cannot be discarded.

Differences between *Daphnia* clones may be dependent on the genotype and life history of these organisms [55,66]. The *D. gessneri* clone was isolated from the Apertado Reservoir, located in Mucugê (BA), where there are no reported occurrences of cyanobacterial blooms. The *D. laevis* clone (IB) was isolated from Ibirité Lagoon (MG), where phytoplankton is dominated by *Microcystis* spp. [67]. The *D. laevis* clone (RD) was isolated from Lake Jacaré (Rio Doce State Park, MG) where *R. raciborskii* has already been reported to occur [68]. Cyanobacteria dominating environments for long periods can act as a natural selection force on *Daphnia* populations, with genotypes with higher growth and reproduction rates being favored [69]. Thus, the differences in sensitivity among *Daphnia* clones to cyanobacteria may be a selective factor, determining the composition of zooplankton, favoring the competitive ability of some tolerant species over other sensitive species [50].

In conclusion, we found adverse effects of toxic *R. raciborskii* CYLCAM-1 on *Daphnia* fitness, even when exposed to a diet without food restriction. The results of the different experimental designs provided evidence that in spite of the constant nutritious food, toxicity still remains as a factor that impairs feeding rates and reproduction. However, species responded differently, with *D. laevis* clones being much more tolerant than *D. gessneri* to a lack of nutritious food. *Daphnia* clones that showed a high clearance rate (i.e., continued feeding even in diets with high cyanobacterial ratios) appeared to be less tolerant to exposure to CYLCAM-01, while clones that reduced their clearance rate in the presence of cyanobacteria were more tolerant and seemed to avoid ingesting filaments, being able to coexist with *R. raciborskii* in conditions where dominance of this cyanobacteria occurs. These are important results which show that in nature different species will have different fitness responses to cope with cyanobacterial blooms.

In a scenario with climate change and an increased dominance of cyanobacteria, the zooplankton community may suffer alterations of its composition due to different levels of sensitivity among organisms. In the natural environment, a reduced predation of zooplankton on phytoplankton may favor the dominance of toxic cyanobacteria, causing the extinction of sensitive species and reduction in diversity of aquatic ecosystems. Studies investigating behavioral resistance (such as rejecting filaments) or physiological tolerance to STX-producing strains are needed for elucidation on the feeding selectivity and ecophysiology of different *Daphnia* clones.

## Figures and Tables

**Table 1:** Biomass (as mg C L^−1^ and %) and STX concentrations in the life table assays without nutritional restriction and with variable proportions of cyanobacteria and nutritious food, and in the grazing assay with clones of *D. laevis* and *D. gessneri*.

Assay	Date	Cyanobacteria Concentration (mg C L^−1^)	Green Algae Concentration (mg C L^−1^)	% Nutritious Food	STX (ng/L)
1. Life table (Constant nutritious food)	16 May 2017	0.00	0.40	100.0	---
		0.50	0.40	44.4	1.5
		1.00	0.40	28.6	3.0
		1.50	0.40	21.1	4.5
2. Life table (Variable nutritious food)	12 March 2018	0.00	1.00	100.0	---
		0.25	0.75	75.0	5.0
		0.50	0.50	50.0	9.9
		0.90	0.10	10.0	15.2
3. Grazing (Variable nutritious food)	16 February 2018	0.00	1.00	100.0	---
		0.25	0.75	75.0	7.2
		0.50	0.50	50.0	14.4
		0.90	0.10	10.0	25.9
4. Grazing (Variable nutritious food)	3 May 2018	0.00	1.00	100.0	---
		0.25	0.75	75.0	3.3
		0.50	0.50	50.0	6.6
		0.90	0.10	10.0	11.9

**Table 2:** LC_50_ and EC_50_ values and 95% confidence intervals (in brackets) of *D. laevis* (IB), *D. laevis* (RD) and *D. gessneri* in the life table assay 1, with constant nutritious food. The symbols (−) indicate that it was not possible to calculate the estimates for the species.

Species	LC_50_ (mg C L^−1^)	EC_50_ (mg C L^−1^)
*D. laevis* (Ibirité)	−	1.24 (1.02–1.52)
*D. laevis* (Rio Doce)	−	0.98 (0.82–1.21)
*D. gessneri*	1.33 (0.75–1.92)	1.68 (1.47–1.92)

**Table 3:** Results of two-way ANOVA for age at first reproduction, fecundity and total offspring in assay 1, with constant nutritious food.

Factor	df	F	*p*
**Age at first reproduction**			
Species	2	35.42	<0.0001
Treatment	3	3.32	0.02
Species × treatment	6	0.74	0.60
**Fecundity**			
Species	2	37.04	<0.00
Treatment	3	15.30	<0.001
Species × treatment	6	1.87	0.09
**Total offspring**			
Species	2	67.35	<0.001
Treatment	3	15.30	<0.001
Species × treatment	6	1.87	0.09

**Table 4:** LC_50_ and EC_50_ values and 95% confidence intervals (in brackets) of *D. laevis* (IB), *D. laevis* (RD) and *D. gessneri* in life table assay 2, with variable proportions of cyanobacteria and nutritious food. The symbol (–) indicates that it was not possible to calculate the estimates for the species.

Species	LC_50_ (mg C L^−1^)	EC_50_ (mg C L^−1^)
*D. laevis* (Ibirité)	–	0.81 (0.23–0,95)
*D. laevis* (Rio Doce)	–	0.89 (0.76–0.97)
*D. gessneri*	0.25 (0.17–0.34)	0.56 (0.53–0.61)

**Table 5:** Results of the two-way ANOVA for age at first reproduction, fecundity and total offspring in assay 2, with variable proportions of cyanobacteria and nutritious food.

Factor	df	F	*p*
**Age at first reproduction**			
Species	2	2.24	0.11
Treatment	3	20.45	<0.001
Species × treatment	6	2.19	0.04
**Fecundity**			
Species	2	5.87	0.003
Treatment	3	38.33	<0.001
Species × treatment	6	7.54	<0.001
**Total offspring**			
Species	2	0.68	0.50
Treatment	3	36.97	<0.001
Species × treatment	6	6.05	<0.001

## Data Availability

The data presented in this study are available on request from the corresponding author.
